# The free energy of locking a ring: Changing a deoxyribonucleoside to a locked nucleic acid

**DOI:** 10.1002/jcc.24692

**Published:** 2017-01-19

**Authors:** You Xu, Alessandra Villa, Lennart Nilsson

**Affiliations:** ^1^ Department of Biosciences and NutritionKarolinska InstitutetHuddingeSE‐141 83Sweden

**Keywords:** DNA, ribose, LNA, free energy perturbation, MD simulation, bridged ring

## Abstract

Locked nucleic acid (LNA), a modified nucleoside which contains a bridging group across the ribose ring, improves the stability of DNA/RNA duplexes significantly, and therefore is of interest in biotechnology and gene therapy applications. In this study, we investigate the free energy change between LNA and DNA nucleosides. The transformation requires the breaking of the bridging group across the ribose ring, a problematic transformation in free energy calculations. To address this, we have developed a 3‐step (easy to implement) and a 1‐step protocol (more efficient, but more complicated to setup), for single and dual topologies in classical molecular dynamics simulations, using the Bennett Acceptance Ratio method to calculate the free energy. We validate the approach on the solvation free energy difference for the nucleosides thymidine, cytosine, and 5‐methyl‐cytosine. © 2017 The Authors. Journal of Computational Chemistry Published by Wiley Periodicals, Inc.

## Introduction

Nucleic acid hybridization occurs in a variety of contexts, including biotechnology and therapeutic applications. In particular, it is of interest to design oligonucleotides that bind a DNA or RNA target sequence with high affinity and specificity, for instance using Watson‐Crick base pairing with the target, or by binding as a third strand in the major groove of a target duplex, thus forming a triple helix. To competitively bind an oligonucleotide to a target molecule, the binding affinity needs to be higher than in the prototype (DNA) duplex. This can be achieved using non‐natural nucleotides, for instance with a modified backbone, which can be uncharged as in the case of peptide nucleic acids,^1^ or have a ribose moiety with restricted conformational flexibility as in locked nucleic acid (LNA).^2^, ^3^

**Figure 1:**
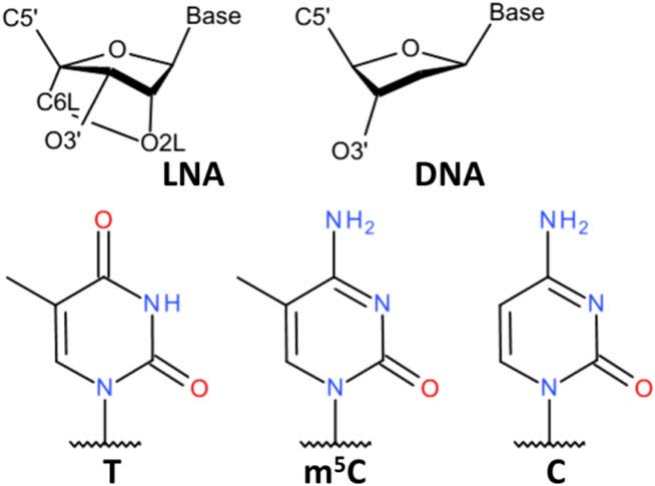
Chemical structures of nucleoside riboses and bases. The first row shows LNA and DNA. The oxymethylene bridge (C6L‐O2L), which locks the sugar pucker in LNA as *C3*′*endo* rather than *C2*′*endo* in DNA, is emphasized. The second row shows the pyrimidine bases thymine, 5‐methyl‐cytidine, and cytidine. [Color figure can be viewed at wileyonlinelibrary.com]

A normal deoxyribose in DNA typically has either the *C2*′*endo* (*south*) or the *C3*′*endo* (*north*) sugar pucker conformation, with *south* being the dominant form under physiological conditions, whereas the oxymethylene bridge in LNA locks the ribose in one pucker. There are two stereoisomers of LNA: β‐D‐LNA which constrains the ribose to be *north* (Fig. 1), and α‐L‐LNA which is constrained as *south*.^4^ The presence of either LNA isomer in an oligonucleotide strand enhances its binding affinity with a DNA or RNA complementary strand as indicated by an increased melting temperature,^4^, ^5^ and the melting temperature is further increased when the fraction of LNA is increased.^3^, ^4^, ^6^ In this study, we focus on β‐D‐LNA (below referred to as LNA) as it is more common in bioassays.

The effect of LNA in a DNA duplex was recently investigated using experimental and designed sequences in molecular modeling.^7^, ^8^, ^9^, ^10^ LNA containing double helixes are under‐wound compared to B‐DNA and the base pair geometries become more A‐like.^7^, ^8^, ^9^ The hydration properties of the grooves are different because of the changed groove dimensions,^7^, ^10^ where LNA duplexes are less hydrated but the network of water molecules is more regular. Binding free energies of duplexes estimated using the method of molecular mechanics generalized Born surface area demonstrate that LNA containing duplexes have lower free energy than all‐DNA duplexes^7^, ^8^; these results are however not quantitative due to the limited accuracy of this methodology.^11^ The physically more rigorous free energy perturbation (FEP) method is expected to yield more accurate results. FEP has been widely applied to nucleic acids, for example, the solvation free energy of nucleobases^12^ and base pairs,^13^ and the binding free energy difference of base mutations in a helical structural context.^13^, ^14^, ^15^ Calculation of free energy differences involving transformations between LNA and DNA nucleotides is more challenging, as this requires the bridging group across the ribose ring to be removed, and breaking bonds is problematic in free energy calculations.^16^, ^17^

The purpose of this study is to develop a protocol to efficiently calculate the free energy difference between LNA and DNA nucleosides. We use both single and dual topology approaches, and because not only the atom types but also the bonded terms are transformed, we break down the perturbation in three steps to investigate the changes in conformation and energy in detail. Based on this, we further propose a one‐step protocol which also performs well, but is more complicated to setup. As LNA‐cytidine is usually synthesized as 5‐methyl‐cytidine (m^5^C_LNA_),^18^ which improves complex stability due to enhanced stacking,^19^ we also calculated the contribution of the methyl group to the solvation free energy.

## Theory and Methods

### Alchemical perturbation

The difference in free energy between two states A and B of a system can be determined using the following expression,^20^

(1) $$\Delta G(A\to B)={{-k}_{B}T\ln{〈\exp\left(-\frac{U_{B}-U_{A}}{k_{B}T}\right)〉}_{A}}_{\;}$$

where *U* is the potential energy, *k*_B_ is the Boltzmann constant, *T* is the temperature, and ${〈\ldots 〉}_{A}$ denotes averaging over state A. If the difference between the two states A and B is small, eq. (1) can be used directly. Alternatively, the difference in free energy can be determined using a coupling parameter approach, where the system potential energy is expressed as a function of coordinates r and coupling parameter *λ* ( λ∈[0,1]),

(2) $$U\left(r,\lambda \right)=U_{0}\left(r\right)+\left(1-\lambda \right)U_{A}\left(r\right)+\lambda U_{B}\left(r\right)\equiv U_{0}\left(r\right)+U'(r,\lambda )$$

Here, *U*_0_ is the potential energy of the environment, that is, the solvent and the common parts between A and B), and *U*_A_ and *U*_B_ are the potential energies of the unique parts of states A and B, respectively. The *λ‐*dependence of the potential energy defines the pathway that connects two states of the system. The difference in free energy between states A and B can be determined by,

(3) $$\Delta G(A\to B)=\sum_{i=1}^{N}\Delta G_{i}{{=-k}_{B}T\sum_{i}\ln{〈\exp\left[-(U_{\lambda_{i+i}}-U_{\lambda_{i}})/k_{B}T\right]〉}_{\lambda_{i}}}_{\;}$$

where *N* is the number of steps necessary to go from A to B, and $\Delta G_{i}$ is evaluated on the intermediate state *λ_i_*. The intermediates are not physical states, that is, the mutation does not have to proceed along a chemical path, so this is called alchemical perturbation. For simplicity we introduce the notation *U_λi_* ≡ *U*(**r**,*λ_i_*).

We used the Bennett Acceptance Ratio (BAR) method^21^ to obtain the free energy differences [eq. (3)], because it has been shown to be very efficient.^22^ BAR has been described in detail in the literature,^21^, ^22^ so here we only give a brief outline of the main equations. According to BAR the difference in free energy in eq. (3) is given by:

(4) $$\Delta G_{i}=k_{B}T\ln\frac{{〈f(U_{\lambda_{i}}-U_{\lambda_{i+1}}+C)〉}_{\lambda_{i+1}}}{{〈f(U_{\lambda_{i+1}}-U_{\lambda_{i}}-C)〉}_{\lambda_{i}}}+C$$

where *f* is the Fermi function,

(5) $$f\left(x\right)=\frac{1}{1+e^{x/k_{B}T}}$$

and *C* is an energy offset related to the partition functions of states $\lambda_{i}$ and $\lambda_{i+1}$. Bennet showed that the free energy in eq. (4) is obtained when the following condition is fulfilled^21^:

(6) $${〈f(U_{\lambda_{i}}-U_{\lambda_{i+1}}+C)〉}_{\lambda_{i+1}}={〈f(U_{\lambda_{i+1}}-U_{\lambda_{i}}-C)〉}_{\lambda_{i}}$$

Inserting eq. (6) into eq. (4) we get ΔG=C, and we can obtain C by iteratively solving eq. (4). Sufficient overlap^22^ between the distributions of the energy differences ${\Delta U}_{\lambda_{i}}^{fw}=(U_{\lambda_{i+1}}-U_{\lambda_{i}}){|}_{\lambda_{i}}$ and ${\Delta U}_{\lambda_{i}+1}^{bw}=(U_{\lambda_{i}}-U_{\lambda_{i+1}}){|}_{\lambda_{i}+1}$ (here ${|}_{\lambda_{i}}$ means the trajectory of window $\lambda_{i}$) is essential for the convergence of this procedure.

**Scheme 1:**
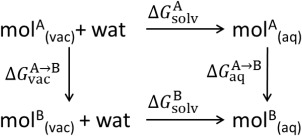
Thermodynamic cycle for the determination of the solvation free energy difference between two molecules. $\Delta G_{solv}^{A}$ and $\Delta G_{solv}^{B}$ are the solvation free energies for molecules A and B, and $\Delta G_{vac}^{A\to B}$ and $\Delta G_{aq}^{A\to B}$ are the free energy differences of mutating molecule A into B in vacuum and solution, respectively.

The solvation free energy $\Delta G_{solv}$ is the work required to transfer a molecule from vacuum into solution. To calculate the relative solvation free energy ${\Delta G}_{solv}^{B}-{\Delta G}_{solv}^{A}$, we use the thermodynamic cycle in Scheme 1. The difference in solvation free energy between two molecules is calculated using

(7) $$\Delta \Delta G_{solv}(A\to B)=\Delta G_{solv}^{B}-\Delta G_{solv}^{A}=\Delta G_{aq}^{A\to B}-\Delta G_{vac}^{A\to B}$$

**Figure 2:**
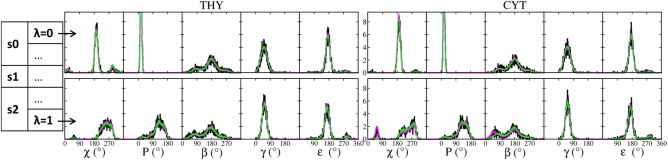
Conformational distributions of thymidine and cytidine in the transformation LNA→DNA. The graph shows the glycosidic torsion (*χ*), sugar pucker (*P*) and backbone torsions *β*, *γ*, and *ε*, in the initial (*λ*_s__0_ = 0, LNA) and final (*λ*_s__2_ = 1, DNA) states (Scheme 2). The distributions in magenta and green are summed from five replicates using single and dual topology, respectively. For comparison, the distributions sampled from 200 ns standard MD simulations of regular LNA and DNA nucleosides are shown in black. The boxes on the left indicate the states from which the distributions were sampled. [Color figure can be viewed at wileyonlinelibrary.com]

### Computational details

#### MD simulation

The CHARMM36 force field for nucleic acids^23^, ^24^ and modified nucleotides^25^ was used. The previously published parameters for LNA^10^ have been slightly modified: (1) the methylene hydrogen type HN8 was used for atom H6′ and H6″, instead of the old type HN1, which is for amino hydrogen; (2) the amplitudes of two terms associated with torsion *γ*, “ON5 CN8B CN7 CP2” and “HN8 CN8B CN7 CP2,” were set to zero. Those dihedral terms were previously covered by a generic term *‐CN8B‐CN7‐* (* is any atom type) and were not explicitly parameterized for LNA; in a pyrimidine nucleoside these terms are not very important as the O5′‐H hydroxyl group, which is the only group whose orientation depends on *γ*, cannot easily form any intramolecular interactions.

The FEPs were performed on nucleosides, and both single and dual topologies were built. In single topology each atom in one state has a counterpart with the same coordinates in the other state, and the atoms that are unique in one state are dummies in the other state.^26^ In dual topology, all atoms, for which the non‐bonded parameters, either charge or Lennard‐Jones (L‐J), differ between the two states, are represented by separate atoms in each state. The CHARMM PERT and BLOCK modules^27^ were used for the single and dual topology free energy simulations, respectively.

The structure was energy minimized using 50 steps steepest descent, followed by 100 steps Adopted‐Basis Newton‐Raphson.^28^ Then, the structure was solvated in TIP3P water^29^ using periodic boundary conditions (PBC), with the shortest distance between a box face and the solute being 8 Å. A cubic box (∼1910 atoms) and a rhombic dodecahedron (∼1260 atoms) were built for each system. The cubic box was used for all dual topology (BLOCK) and standard simulations, which were run on graphical processing units (GPUs), and the rhombic dodecahedron was only used for single topology (PERT) simulations, which were run on CPUs. A lookup table^30^ was used for the non‐bonded interactions between non‐mutated atoms in CPU jobs. As no phosphate was included, the systems are not charged and no ions were added. The particle mesh Ewald method^31^ was applied for long range electrostatic interactions, with a direct space cutoff of 9 Å, and a force switch function was used to switch the van der Waals interactions over the range 8–9 Å. For *in vacuo* simulations we used infinite cutoffs as no solvent and no PBC was present. All covalent bonds involving hydrogen atoms were constrained using the SHAKE algorithm.^32^ All simulations were performed with the program CHARMM,^27^ together with the OpenMM interface for runs utilizing GPUs.^33^ The simulations were performed at 298 K in the NPT ensemble using Langevin dynamics with a friction coefficient of 5 ps^−1^. The leap‐frog integrator was used with a 2 fs time step. After 10 ps equilibration, each window was run for 10 ns (20 ns for some runs in dual topology; Table 1) in both solution and vacuum for each system. Five independent runs with the same starting structure but different initial velocity assignments were performed for each system.

**Table 1:** Systems used in free energy perturbation.

			Step 0		Step 1		Step 2		
Nt.	Transformation	Topology	Method	# *λ*	Method	# *λ*	Method	# *λ*	Total # *λ*
T/C	LNA→DNA	Single	*K_*θ*_* & *K_*φ*_* scaling	5^a^	PERT	11^c^	*K_*θ*_* & *K_*φ*_* scaling	11^d^	27
		Dual		11^b^	BLOCK	11^c^		15^e^	37
m^5^C	LNA→DNA	Dual		11^b^	BLOCK	11^c^		15^e^	37
	Meth→Hydr	Single	–		PERT	11^c^	–		11

a *λ*s: 0, 0.01, 0.05, 0.2, 1.

b *λ*s: 0, 0.005, 0.01, 0.03, 0.05, 0.1, 0.2, 0.4, 0.6, 0.8, 1.

c *λ*s: 0, 0.1, 0.2, 0.3, 0.5, 0.7, 0.8, 0.9, 0.99, 0.999, 1.

d *λ*s: 0, 0.4, 0.6, 0.8, 0.9, 0.95, 0.97, 0.98, 0.99, 0.995, 1.

e *λ*s: 0, 0.2, 0.4, 0.6, 0.8, 0.85, 0.9, 0.93, 0.95, 0.97, 0.98, 0.99, 0.995, 0.999, 1.

For all systems 5 independent replicates are simulated, with 10 ns sampling time for each *λ* (20 ns for step0 and step1 in dual topology).

#### Analysis

MD snapshots saved every 2 ps were analyzed using CHARMM. The potential energies $U_{\lambda_{i+i}}$ and $U_{\lambda_{i}}$ were extracted from each trajectory using the MSCALE module^34^ in post‐processing. The free energy difference was calculated by iteratively solving eq. (4) with a perl script (G. König and S. Boresch, private communication) based on the result that Δ*G* = *C* when the iterations converge. At each iteration, the current value for *C* is inserted into the right‐hand side of eq. (4), which is evaluated to give a new value for *C*. The iterations start with the initial guess *C* = 0.0, and continue until *C* changes by < 10^−5^ kcal/mol. This procedure is very fast, and in practice <100 rounds are sufficient to get convergence. The overlap between the normalized distributions of ${\Delta U}_{\lambda_{i}}^{fw}$ and ${\Delta U}_{\lambda_{i}+1}^{bw}$ is calculated according to eq. (21) in Ref. [ 22]. The standard deviation of each $\Delta G_{i}$ was calculated from five independent replicate simulations.

The glycosidic torsion (*χ*) is defined by the dihedral O4′‐C1′‐N1‐C2 for both thymidine and cytidine, and the conformation is termed *anti* when 170° < *χ* < 300° (low *anti*: *χ* < 220°; high *anti*: *χ* > 270°) and *syn* when 30° < *χ* < 90°. The sugar pucker is defined by the pseudorotation phase angle (*P*)^35^ which is a combination of five ring torsions, and it is denoted as *north* (−90° < *P* ≤ 90°) and *south* (90° < *P* ≤ 270°). Other backbone torsions that were analyzed in this study are defined as *β*: H5T‐O5′‐C5′‐C4′, *γ*: O5′‐C5′‐C4′‐C3′, and *ε*: C4′‐C3′‐O3′‐H3T.

The potential of mean force (PMF) along the dihedral angle *χ* was calculated using umbrella sampling with a harmonic biasing potential with force constant of 250 kcal/mol/rad^2^. We used 72 independent simulation windows, in which the reference dihedral angle varied from 0 to 360° in 5° intervals. Each window was run for 2 ns. Finally, the bias potential was removed and the PMF as a function of *χ* was calculated with the weighted histogram analysis method.^36^

## Results and Discussion

A three‐step protocol was designed to mutate the locked ribose of an LNA into the deoxyribose of a DNA nucleoside. In the following section, we describe the protocol in detail using both single and dual topologies. Then, we check whether the initial and final states in the hybrid topologies sample the identical conformational space as in standard simulations of LNA and DNA nucleosides, and whether the end state of one step is identical to the initial state of the following step in the three‐step protocol. We also discuss the different free energy contributions in steps using single and dual topologies. In the next section, we investigate the performance of the three‐step approach and propose an alternative single step approach. Finally, we apply the three‐step protocol in a case study: transforming m^5^C_LNA_ to C_DNA_.

### Three‐step protocol

#### System setup

**Scheme 2:**
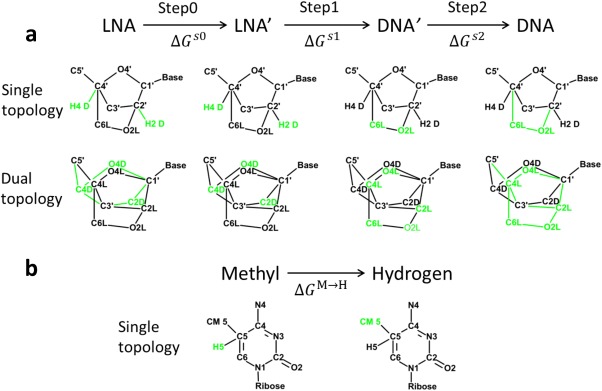
The transformation process and the topologies used in each state, showing a) the ribose change from state LNA to state DNA and b) the base change from m^5^C to C. In each sketch, the real atoms and the bonded terms are in black, whereas the dummy atoms (with zero electrostatic and L‐J interactions) and dummy bonded terms (with zero angle and dihedral but original bond force constants) are green. For clarity, hydrogen atoms are omitted, except for H2D/H4D and H5 in single topology. [Color figure can be viewed at wileyonlinelibrary.com]

Atom types and partial atomic charges in the sugar moiety of DNA or LNA in the CHARMM force field are independent of the nature of the base, so the same hybrid topology for the sugar was used for both thymidine and cytidine (Scheme 2). Oxymethylene atoms, which are unique for the LNA are named C6L, H6L, H6′L, and O2L. Ribose atoms (C1′, C3′, C5′, and the connected exocyclic atoms) that have identical L‐J parameters and charges in the initial and final states were treated as common atoms, while ribose atoms (C2′, C4′, O4′, and the connected exocyclic atoms) that differ in charge or L‐J parameters between initial and final states were treated differently in single and dual topologies. In a strict single topology setup, it is problematic to perturb a bond which is constrained by SHAKE in one state, but is un‐constrained in the other. For the LNA to DNA change, this could be the case for H2′/O2L (attached to C2′), and H4′/C6L (attached to C4′), with SHAKE applied to C2′‐H2′ and C4′‐H4′ in DNA, but not to C2′‐O2′ and C4′‐C6′ in LNA. To avoid this complication, we deviated from the strict single topology, and treated H2′ and H4′ as unique for DNA (and renamed them to H2D and H4D), so that we used dual coordinates for these hydrogen atoms, whereas the other ribose atoms had the same coordinates in the initial and final states. In dual topology the LNA atoms O4L, C4L, C2L, and H2′L are separate from the DNA atoms O4D, C4L, C2D, and H2D; this requires four more atoms than in single topology.

**Figure 3:**
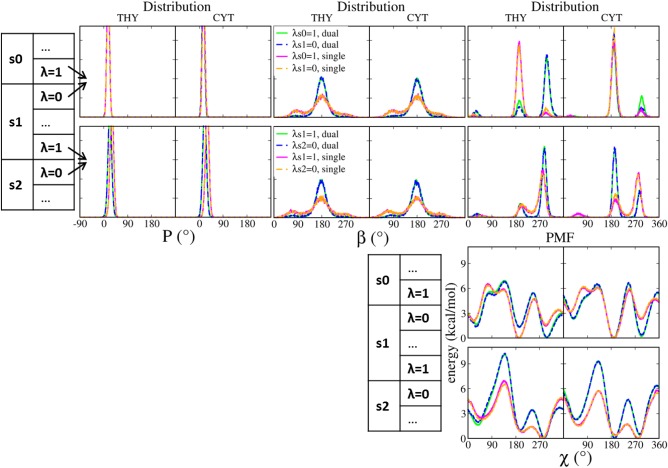
Distributions of *P*, *β*, and *χ*, and the PMF of *χ* for the intermediate states (LNA′ and DNA′) as represented by the end of *s*0/beginning of *s*1, and end of *s*1/beginning of *s*2, in the transformation LNA→DNA. The distributions are summed from five runs whereas the PMFs were calculated using umbrella sampling. Green and blue: dual topology; magenta and orange: single topology. The boxes on the left indicate the states from which the conformations were sampled. [Color figure can be viewed at wileyonlinelibrary.com]

As the LNA nucleoside has an extra oxymethylene group, which fixes the sugar in the *north* conformation, both nonbonded and bonded energy terms are perturbed in the transformation to a DNA nucleoside. The transformation started from state LNA, and in the first step (s0) the DNA bonded energies were added. However, to avoid having freely moving DNA atoms in the initial state (LNA) and similarly for the LNA atoms in the final state (DNA), all the bond terms were present at full strength in all states. Therefore, only angle and dihedral energy were actually scaled in s0:

(8) $$U^{\text{s}0}\left(\lambda \right)=U_{\text{a},\text{d}}^{\text{D}}(\lambda K_{\theta },\;\lambda K_{\varphi })+U_{b}^{\text{D}}+U_{bd}^{\text{L}}+U_{nb}^{\text{L}}+U_{0}^{env}$$

where the superscripts D and L denote DNA and LNA, subscripts bd and nb stand for bonded and nonbonded energies while b, a, and d specifically denote bond, angle and dihedral respectively; $K_{\theta }$ and $K_{\varphi }$ are force constants and amplitudes of angles and dihedrals, respectively, and $U_{0}^{env}$ includes all terms of the other, unchanged, atoms. The first term of the right‐hand side in eq. (8) corresponds to *U*′ and the other terms correspond to *U*_0_ in eq. (2).

The next state LNA′ has LNA nonbonded interactions and both DNA and LNA bonded interactions. In the second step (s1), the nonbonded parameters were transmuted from LNA atoms to DNA, while all bonded parameters were unchanged. In this step, the *λ*‐scaled nonbonded energies between initial and final states were handled by FEP modules PERT or BLOCK in CHARMM. At the end of this step, DNA′ is an intermediate which has DNA charges and L‐J parameters but both DNA and LNA bonded energies. The potential energy in *s*1 is

(9) $$U^{\text{s}1}\left(\lambda \right)={\left(1-\lambda \right)U_{nb}^{\text{L}}+\lambda U_{nb}^{\text{D}}+U}_{bd}^{\text{L}}+U_{bd}^{\text{D}}+U_{0}^{env}$$

Particularly in single topology, when the atom types are transmuting, the bonded force field parameters of mutated atoms are also changed. Thus, eq. (9) in single topology can be expressed as

(10) $$U^{\text{s}1}\left(\lambda \right)={{\left(1-\lambda \right)(U}_{bd,mut}^{\text{L}}+U_{nb}^{\text{L}})+\lambda (U_{bd,mut}^{\text{D}}+U_{nb}^{\text{D}})+U}_{bd}^{\text{L}}+U_{bd}^{\text{D}}+U_{0}^{env}$$

where the subscript mut denotes the bonded terms involving mutated atoms.

In the final step (s2), from DNA′ to DNA, the LNA angle and dihedral energies were turned off, and the final state DNA has all DNA energy terms and LNA bond terms. The energy in this step is

(11) $$U^{\text{s}2}\left(\lambda \right)=U_{a,d}^{\text{L}}(\lambda K_{\theta },\;\lambda K_{\varphi })+U_{b}^{\text{L}}+U_{bd}^{\text{D}}+U_{nb}^{\text{D}}+U_{0}^{env}$$

The total transformation energy from LNA to DNA through the three steps thus is:

(12) $$\Delta G(L\to D)=\Delta G^{\text{s}0}+\Delta G^{\text{s}1}+\Delta G^{\text{s}2}$$

The transformations in s0 and s2, which only involve scaling of bonded force field parameters, were performed as standard MD simulations; the relevant parameters were scaled by *λ* in the parameter file at the beginning of the simulation using the CHARMM internal variable facility. The DNA and LNA specific atoms were assigned unique atom types to allow scaling of just these particular angle and dihedral terms. It is also possible to scale all angle and dihedral energies of LNA or DNA using for instance the BLOCK module. However, the current scheme has two advantages: a normal MD simulation is used, and it is possible to select exactly which individual parameters to scale so the energy change in the transformation is reduced.

The transformation from m^5^C_LNA_ to C_DNA_ requires one more step: the change of the 5‐methyl group into hydrogen on the base (Fig. 1). Here, single topology was used: the base atoms had the same coordinates in the two states, except for H5 and CM5, to avoid the constraint issue. The perturbation methyl group to hydrogen atom (M→H) was performed independently from the sugar transformation. The difference in solvation free energy of m^5^C_LNA_ to C_DNA_ was calculated both with LNA and DNA sugars. Thus in addition to C_LNA_→C_DNA_ described above, three more transformations were performed: m^5^C_LNA_→m^5^C_DNA_, m^5^C_LNA_→C_LNA_ and m^5^C_DNA_→C_DNA_. For m^5^C_LNA_→m^5^C_DNA_ only the dual topology method was used.

#### Conformational distributions

The main conformational difference between LNA and DNA is the sugar pucker: LNA sugar is in a rigid *north* conformation, whereas DNA sugar is flexible with ∼70% population in *south*.^37^ The torsion *χ* is affected by the sugar conformation^37^; thus, it is also different between DNA and LNA. To compare the sampling of the hybrid topologies and the standard topologies for LNA and DNA, we collected distributions of the sugar pucker and key dihedral angles from the five replicates of the initial and final states of the FEP calculations and from 200 ns standard simulations of thymine and cytidine nucleosides with LNA and DNA sugars (Fig. 2).

**Figure 4:**
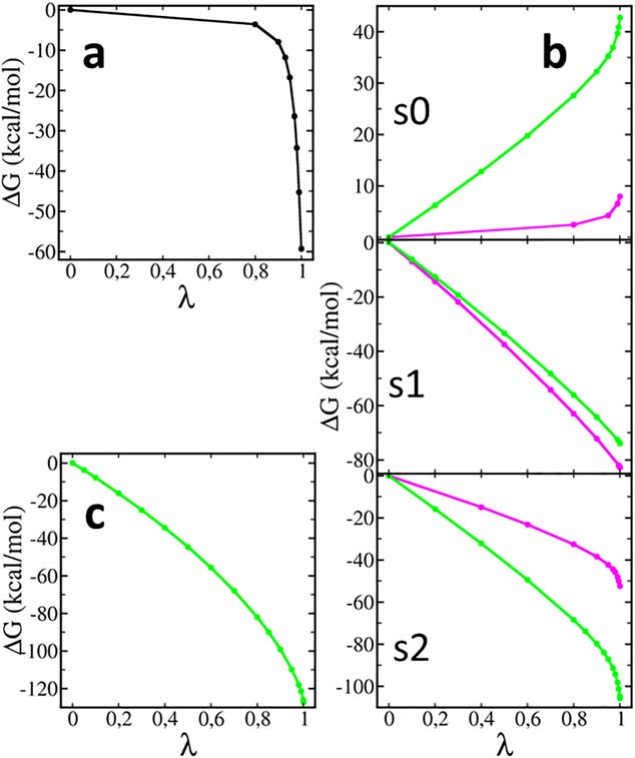
Accumulation of the free energy change as a function of the scaling factor *λ*. a) The original trial: Δ*G* change with turning off the harmonic distance restraints between O2L and C6L. b) The three‐step protocol: *s*0, Δ*G* change while turning on the DNA angle and dihedral energy; *s*1, Δ*G* change with charge and L‐J transformation between LNA and DNA; *s*2, Δ*G* change while turning off the LNA angle and dihedral energy, using single (magenta) and dual (green) topology. c) The one‐step protocol: Δ*G* change while simultaneously turning on DNA bonded energy, transforming charges and L‐J parameters, and turning off LNA bonded energy. The error bars are comparable to the line thickness. [Color figure can be viewed at wileyonlinelibrary.com]

In both single and dual topologies, the sugar puckers were in *north* in the initial state and mainly in *south* in the final state, in agreement with the distributions observed from the standard simulations. The *χ* distributions also agreed well between the FEP and standard simulations, with *χ* mainly as low *anti* in state LNA and as wide *anti* in state DNA. Even the minor fraction of *syn* and high *anti* in LNA and *syn* in DNA were sampled in both topologies. Small sampling discrepancies are seen for *χ* of C in the final state, in which the dual topology somewhat under‐sampled *syn*. The backbone torsions *β*, *γ*, and *ε* have almost the same distributions in LNA and DNA, and at the end states the distributions were consistently reproduced in both the single and dual topologies. The slightly skewed distribution of *β* in dual topology is due to the attraction between 5′‐OH and base O2 when *χ* is *syn*,^37^ which is only present in pyrimidine nucleosides. Overall both single and dual topologies reproduced LNA and DNA conformations in the initial and final states.

An important requirement of the three‐step protocol is that the final state of one step and the initial state of the following step sample the same conformational space (i.e., the identity between *λ_s_*_0_ = 1 and *λ_s_*_1_ = 0, and *λ_s_*_1_ = 1 and *λ_s_*_2_ = 0). Of the torsions (*χ*, *P*, *β*, *γ*, and *ε*) that were involved in the transformation, the largest difference in the conformational distribution between the LNA and DNA was observed for *χ* and *P* (Fig. 2), and we selected *χ* and *P* together with *β* to inspect the conformations in the intermediate states.

The sugar pucker in LNA′ and DNA′ states shows almost the same preference for *north* as in LNA (Fig. 3), and converted to *south* when LNA angle and dihedral energies were removed in *s*2 (Fig. 2). The backbone torsions have the same distributions in both LNA′ and DNA′ states, but with dual topology the distributions were more narrow compared to the end states. The torsion *χ* varied among *syn*, low and high *anti* in all states of both topologies. The torsion distributions from adjacent states (LNA′: *λ_s_*_0_ = 1/*λ_s_*_1_ = 0; DNA′: *λ_s_*_1_ = 1/*λ_s_*_2_ = 0) overlapped perfectly, except for *χ* in LNA′ in dual topology (Fig. 3). To further probe this, we computed PMF along *χ* for those adjacent states. The PMFs are indeed identical between adjacent states within either topology, and the positions of the energy minima are consistent with the *χ* distributions (Fig. 3). However, in the PMFs the barriers between low and high *anti* in state LNA′ are about 6 kcal/mol, which is higher than in state DNA′. Because of this, the conformations of χ in LNA′ especially for cytidine did not interconvert in first 10 ns. With the extended 20‐ns sampling time for *s*0 and *s*1 in dual topology the conformational interconverting was observed, although it was still not long enough to obtain exactly the same distribution for *λ_s_*_0_ = 1 and *λ_s_*_1_ = 0. The higher barrier observer in the dual topology may be due to the presence of both DNA and LNA bonded terms in the intermediate states.

**Figure 5:**
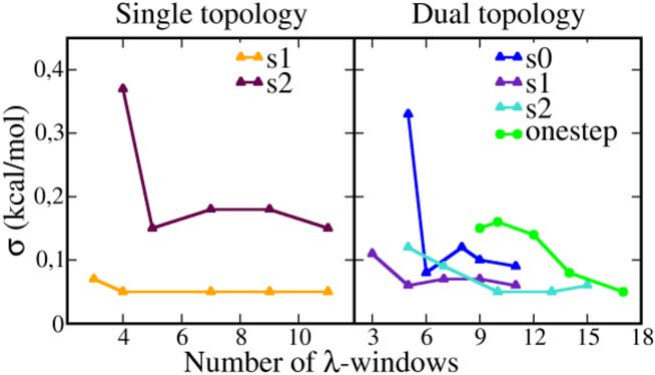
The standard deviation (*σ)* from five replicates as a function of the number of *λs* used in the BAR calculation for thymidine. Step *s*0 in single topology was omitted. [Color figure can be viewed at wileyonlinelibrary.com]

#### Free energy changes

In the previous sections, we have shown that the hybrid topologies reproduce the correct conformations for physical states and consistently sample the intermediate states. Here, we analyze in detail the free energy contribution of each step in the case of single or dual topology, *in vacuo* and in solution. Overall the results for thymidine and cytidine are very similar (Table 2).

**Table 2:** The stepwise transformation free energy (kcal/mol) of converting LNA to DNA for thymidine and cytidine using single and dual topology.

		Single topology			Dual topology		
		$\Delta G_{aq}^{}$	$\Delta G_{vac}^{}$	$\Delta \Delta G_{}^{}$	$\Delta G_{aq}^{}$	$\Delta G_{vac}^{}$	$\Delta \Delta G_{}^{}$
**T**	*s*0	7.93 (0.03)	7.90 (0.07)	0.03 (0.08)	41.94 (0.09)	42.96 (0.06)	−1.02 (0.11)
	*s*1	−82.81 (0.05)	−84.06 (0.01)	1.25 (0.05)	−73.90 (0.03)	−76.55 (0.02)	2.65 (0.04)
	*s*2	−52.44 (0.15)	−52.33 (0.06)	−0.11 (0.16)	−105.73 (0.06)	−105.36 (0.18)	−0.37 (0.19)
**C**	*s*0	7.89 (0.04)	7.96 (0.03)	−0.07 (0.05)	41.79 (0.11)	41.46 (0.04)	0.33 (0.12)
	*s*1	−75.60 (0.07)	−77.14 (0.00)	1.54 (0.07)	−67.31 (0.02)	−69.74 (0.02)	2.43 (0.03)
	*s*2	−52.25 (0.07)	−52.09 (0.05)	−0.16 (0.09)	−104.56 (0.09)	−103.59 (0.06)	−0.97 (0.11)

Reported values are average of five runs with the standard deviations (*σ*) in parenthesis.

In single topology, the total free energy was dominated by $\Delta G^{s1}$, where all nonbonded and bonded parameters for ring atoms were transmuted [eq. (10)]. The absolute value of ${\Delta \Delta G}^{s1}$ is larger than $\Delta \Delta G_{}^{s0}$ and ${\Delta \Delta G}^{s2}$, which are negligible. ${\Delta \Delta G}^{s1}$ reflects the free energy difference between LNA′ and DNA′, which responds to the changed solvent interaction and ring strain. The remaining angle and dihedral terms were turned on/off in *s*0 and *s*2 [eqs. 8 and 11], where $\Delta G^{s2}$ is larger than $\Delta G^{s0}$. Neither the conformational change nor $\Delta \Delta G_{}^{s0}$ is significant in the LNA→LNA′ transformation, *s*0 thus is unnecessary in single topology, and LNA′ is in practice equal to LNA. The contribution of $\Delta \Delta G_{\;}^{s2}$, which reflects the difference between restrained and unrestrained sugar conformations, is quite small for a mononucleoside, but it may be more important in an helical context where the energy cost of changing the sugar pucker may be larger.

In dual topology, $\Delta G^{s1}$ contains only charge and L‐J contributions [eq. (9)] so it is 10% smaller than in single topology, but $\Delta \Delta G^{s1}$ is still the dominant term and even larger (Table 2). In contrast to single topology, the conformational change in dual topology (Fig. 3) in *s*1 is only due to the nonbonded transformation, which in a non‐ring topology would be sufficient to account for the solvation effect.^16^, ^26^ For a bridged topology, the ring conformation is however confined by bonds from the atoms of both states, so $\Delta \Delta G^{s1}$ contains a contribution also from this conformational restriction. Therefore, *s*0 and *s*2 are necessary in the dual topology. $\Delta G^{s0}$ and $\Delta G^{s2}$ represent the angle and dihedral energies for DNA and LNA atoms respectively, and $\Delta \Delta G^{s0}$ and $\Delta \Delta G^{s2}$ are the corresponding free energies of restriction on states LNA and DNA.

The total solvation free energy differences, $\Delta \Delta G_{solv}^{L\to D}$, for thymine and cytidine are about 1.2 and 1.3 kcal/mol in single and 1.3 and 1.8 kcal/mol in dual topology, respectively (Table 3). It means that an LNA nucleoside is 1–2 kcal/mol more favorable than DNA in aqueous solution. $\Delta \Delta G_{solv}^{L\to D}$ in dual topology is larger than in single, but given the standard deviation (∼0.3 kcal/mol) the values are indistinguishable. Experimental solvation free energies for LNA and DNA nucleosides are not available, but we can compare our results with the hydration free energy ( $\Delta G_{h}^{0}$) of linear and cyclic ethers.^38^ For example, inserting an oxygen to tetrahydrofuran (making it 1,4‐dioxane) decreases $\Delta G_{h}^{0}$ by 1.58 kcal/mol, while inserting a ‐OCH2‐ group in linear ethers decreases $\Delta G_{h}^{0}$ by 1.0–1.7 kcal/mol. The solvation free energy difference of 1.0–1.6 kcal/mol between the locked ribose and deoxyribose is reasonable.

**Table 3:** The solvation energy difference, $\Delta \Delta G_{solv}^{L\to D}$, of converting LNA to DNA (kcal/mol).

	Single	Dual
**T**	1.17 (0.18)	1.26 (0.22)
**C**	1.31 (0.12)	1.79 (0.17)

Reported values are average of five runs with the standard deviations (*σ*) in parenthesis.

#### Convergence

The simulation at each λ was run for 10 ns. To check the convergence, we calculated the free energy change using each 5 ns interval of the aqueous solution simulations, together with the whole (10 ns) in vacuum (Table 4). We found no substantial difference between the two halves (around 0.1 kcal/mol). For the single topology, the results from 0 to 5 ns and 5 to 10 ns were very close, which suggests that 5 ns might be already sufficient in practice. For dual topology, cytidine had the same $\Delta G_{aq}^{}$ in each 5 ns interval, whereas the fluctuations were larger for thymidine. This suggests that 5 ns sampling, which is not enough for conformational interconversion of the alchemical intermediate state LNA′, is fine for $\Delta G_{aq}^{}$ of cytidine, but for thymidine longer sampling is better.

**Table 4:** The solvation free energy difference $\Delta \Delta G_{solv}^{L\to D}$ (kcal/mol) using different amounts of sampling.

	Single topology		dual topology			
	0–5 ns	5–10 ns	0–5 ns	5–10 ns	10–15 ns	15–20 ns
**T**	1.23 (0.15)	1.15 (0.27)	1.37 (0.40)	1.44 (0.27)	1.13 (0.28)	1.10 (0.33)
**C**	1.37 (0.12)	1.26 (0.29)	1.79 (0.17)	1.77 (0.19)	1.77 (0.20)	1.82 (0.16)

Reported values are average of five runs with the standard deviations (*σ*) in parenthesis.

An atom that is created, and thus appears suddenly near an end state, may have steric clashes with the environment, which causes sampling problems. This can be remedied using soft‐core potentials,^39^ where the energy is bounded when atoms are created.^40^ In this study, all the bonded energy terms are present during the L‐J transformation in *s*1, which ensures that the atoms in the initial and final states occupy almost the same space. This means that the end‐point problem is not severe, and we did not use soft‐core potentials. The LNA groups are more bulky than DNA, and at *λ_s_*_1_ = 1 a small part of the LNA volume is outside the DNA vdW radius so the energy ${〈U_{\lambda <1\;}〉}_{1}$ contains larger L‐J repulsion with the environment than ${〈U_{\lambda =1}〉}_{1}$, which influences the overlap. But this repulsion is finite and the relatively minor problem can be remedied by inserting a few windows (e.g., 0.99, 0.999) near *λ_s_*_1_ = 1.

### Alternative approaches

#### Scaling bonded terms

Simply breaking the “lock,” the C6L‐O2L bond, is an alternative approach that we have also tried. In this original trial, the nonbonded energies were transformed in the first step, and then, the connectivity between C6L and O2L was replaced with a harmonic restraint that was scaled in the second step. The free energy change was however quite steep when the harmonic restraint was close to disappearing (Fig. 4a). Furthermore, because breaking connectivity also removed the corresponding angle and dihedral terms, the Hamiltonian was altered abruptly, which leads to sampling and convergence problems. In the current three‐step protocol, the free energy change in *s*0 and *s*2 (where angle and dihedral terms are scaled) is less steep (Fig. 4b). More closely spaced *λ*‐windows are still required near the final states, due to the harmonic angle terms. Conversely, the relatively poor performance of *s*0 and *s*2 is better than the convergence difficulty encountered when turning off the bond force constant (Fig. 4a), which has also been pointed out previously.^16^, ^26^, ^41^ The probable reason is that when the bond is broken the conformational space is the full extent of the system, which is hard to sample adequately, whereas in the three‐step protocols (with the angle turned off) the range that has to be sampled is confined between 0° and 360°. Also a distinct advantage of using BAR is that it only requires a small energy overlap between adjacent windows,^22^ so that good convergence was achieved even with a smaller number of windows near the end state.

#### Is a one‐step transformation sufficient?

One step is always available for single topology because the bonded terms are converted along with the atom types. This study used dual coordinate for H2D/O2L and H4D/C6L in single topology which required a second step (*s*2). The multi‐step approach allows detailed control over the different (bonded and non‐bonded) transformations. Now it is interesting to know if one step is possible. To implement this in dual topology, the parameters belonging to LNA and DNA need to scale independently. (The dual coordinate containing single topology is a simple case of the dual topology.)

To control the DNA and LNA parameters separately, different atom types are created for LNA and DNA while all the parameters are copied from original atoms, for example, C4L and C4D which keep the same LJ parameters are treated as different atom types in topology file. The three‐step protocol did not need soft‐core because the LJ was transformed before bonded parameters turning off. When they are transformed at the same time, restraints are needed to keep the counterpart atoms close to each other. This is achieved by carefully excluding some angles involving C2′, C3′, and C4′ from scaling so that the ring scaffold is not deformed when the other energy terms of one state are off (force field files in Supporting Information). To minimize the bonded energy changes, most angles involving hydrogens were also excluded.

However, the presence of the LNA scaffold hampers the reproduction of DNA *χ* and pucker distributions. We, therefore, chose to shrink the LNA group when approaching the DNA state. Thus, three bonds, C4L‐C6L, C2L‐O2L, and C6L‐O2L are scaled. For the first two terms, the bond lengths are scaled and constrained by SHAKE, and for the last term the force constant is scaled. The idea is to allow C6L and O2L to remain in the L‐J volume of DNA atoms when the LNA bonded and nonbonded energies disappear. Although the harmonic bond C6L‐O2L was scaled, the sampling space of C6L and O2L was limited because of the restricted conformation by unchanged angles. We experienced numerical problems when bond lengths were made very short, and thus we did not allow the C4L‐C6L and C2L‐O2L bond lengths to become shorter than half their original lengths. The total potential energy of the system thus is similar to eq. (10) of single topology but with some adaption:

(13) $$U^{os}\left(\lambda \right)={\left(1-\lambda \right)U_{nb}^{L}+U_{b_{1},a_{1},d}^{L}\left[\left(1-\lambda \right)K\right]+U_{b_{2}}^{L}\left[\left(0.5-0.5\lambda \right)b\right]+\lambda U_{nb}^{D}+U_{a_{1},d}^{D}(\lambda K)+U}_{b_{0},a_{0}}^{L}+U_{b,a_{0}}^{D}+U_{0}^{env}$$

where subscripts a_1_, b_1_, and b_2_ indicate the scaled bonds and angles, whereas b_0_ and a_0_ are not scaled; *K* is generalized for *K_b_*, *K_θ_* or *K_φ_*, and *b* is for bond length.

A scheme with only 17 *λ*s was used (*λ* = 0, 0.05, 0.1, 0.2, 0.3, 0.4, 0.5, 0.6, 0.7, 0.8, 0.85, 0.9, 0.95, 0.98, 0.99, 0.999, 1). Five simulations of 10 ns are performed for each window *in vacuo* and in solution. The overall solvation free energy differences (Table 5) are in agreement with the three‐step approach, and the values do not depend on which half of the trajectory it used, which indicates that the calculations are well converged. The LNA and DNA conformations are also correctly reproduced in the initial and final states by the hybrid topology (Supporting Information Figure S1).

**Table 5:** The transformation and solvation energy difference (kcal/mol) of converting LNA to DNA using the one‐step protocol and different amounts of sampling.

	0–10 ns			0–5 ns	5–10 ns
	$\Delta G_{aq}^{L\to D}$	$\Delta G_{vac}^{L\to D}$	$\Delta \Delta G_{solv}^{L\to D}$	$\Delta \Delta G_{solv}^{L\to D}$	$\Delta \Delta G_{solv}^{L\to D}$
**T**	−129.77 (0.05)	−130.74 (0.05)	0.97 (0.07)	0.98 (0.15)	0.96 (0.14)
**C**	−122.37 (0.06)	−123.58 (0.05)	1.21 (0.08)	1.15 (0.17)	1.27 (0.09)

Reported values are average of five runs with the standard deviations (*σ*) in parenthesis.

#### Computational efficiency

To test if fewer windows can reach the same precision, we reduced the number of windows used in the BAR calculation in each step, recalculated ΔG and compared the minimal amount of overlap (*O*_min_) between adjacent windows and the standard deviations (*σ*) of the free energies (Supporting Information Table S1 and Fig. 5). The minimal overlap decreased rapidly as the number of *λ*s was reduced, but the standard deviations did not change correspondingly. This is consistent with a previous study,^22^ which shows that 1% overlap in BAR is sufficient to obtain good precision. *σ* does not begin to increase until the number of *λ*s is reduced to less than half of the original number in both topologies (Fig. 5). The minimum number of *λ*s that is required to keep *σ* ≤ 0.2 kcal/mol and *O*_min_ ≥ 1% is 8 (single topology) and 16 (dual topology) for the three‐step protocol, and 9 for the one‐step protocol (Supporting Information Table S1). It is expected that the single topology requires fewer windows than dual topology. The one‐step protocol in dual topology requires fewer windows to reach the same precision as the three‐step, it is however more complicated to setup.

The difference in computational cost between the two topologies is that the single topology does not require *s*0 and the barriers between conformations are lower (Table 1), whereas the dual topology needs longer sampling time near state LNA′. The current implementation of BLOCK with OpenMM in CHARMM allows the simulation on GPU using dual topology, and the 10 ns simulation of a nucleoside in solution (∼1910 atoms) using an NVIDIA GTX780TI GPU takes ∼45 min.

#### m^5^C_LNA_→C_DNA_

**Scheme 3:**
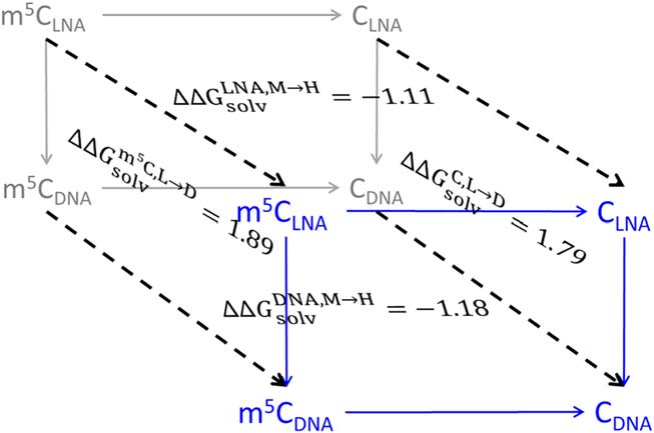
Thermodynamic cycles for the calculation of the solvation free energy difference between m^5^C_LNA_ and C_DNA_. The front and back faces are the transformations in vacuum (gray) and aqueous solution (blue), respectively. Solid arrows represent the calculated alchemical transformations (Tables 3 and 6), and dashed arrows represent the solvation process. The solvation free energy difference for each of the four transformations in Tables 3 and 6 is shown on the face whose edges constitute the corresponding cycle. [Color figure can be viewed at wileyonlinelibrary.com]

The complete transformation for LNA cytidines (m^5^C_LNA_) includes the transformation of the methyl group into a hydrogen atom (M→H) in addition to the LNA→DNA sugar transformation. As the base and ribose are independent atom groups in the force field, their hybrid topologies were built independently (Scheme 2), and the total transformation can be made using LNA→DNA in C or in m^5^C, appropriately combined with M→H in LNA or DNA (Scheme 3, Tables 3 and 6).

**Table 6:** The transformation and solvation free energy difference (kcal/mol) of converting LNA to DNA (dual topology) and Methyl to Hydrogen (single topology) for the m^5^C nucleoside.

Transformation		$\Delta G_{\mathrm{aq}}$	$\Delta G_{\mathrm{vac}}$	$\Delta \Delta G_{\mathrm{solv}}$
LNA→DNA	*s*0	41.67 (0.11)	41.23 (0.03)	1.89 (0.24)
	*s*1	−67.04 (0.04)	−69.57 (0.03)	
	*s*2	−104.45 (0.12)	−103.37 (0.16)	
L: Meth→Hydr		−42.41 (0.02)	−41.30 (0.03)	−1.11 (0.04)
D: Meth→Hydr		−42.86 (0.11)	−41.68 (0.02)	−1.18 (0.11)

Reported values are average of five runs with the standard deviations (*σ*) in parenthesis.

We applied dual topology for LNA→DNA in m^5^C, and for M→H we used single topology with dual coordinates applied for H5 and CM5. The energy associated with the LNA→DNA transformation for m^5^C (1.89 kcal/mol) is close to the values for C, suggesting that the sugar modification is not affected by the base methylation. The M→H transformations in LNA and DNA nucleosides gave very similar results (−1.1 to −1.2 kcal/mol), which means that removing a methyl group (bulky and hydrophobic) from the nucleoside results in a more favorable (more negative) solvation free energy. Our results are comparable with the experimental solvation energy change between benzene and methylbenzene, −1.07 kcal/mol.^38^ The sugar and methyl group transformations act in opposite directions, and the total m^5^C_LNA_→C_DNA_ solvation free energy difference is around 0.7 kcal/mol with a difference between the two paths of only 0.03 kcal/mol:

$${{\Delta \Delta G}_{solv}\left(m^{5}C_{LNA}\to C_{DNA}\right)=\Delta \Delta G}_{solv}^{m^{5}C,L\to D}+\Delta \Delta G_{solv}^{DNA,M\to H}=0.71\;\;kcal/mol$$

or

(14) $${\Delta \Delta G}_{solv}(m^{5}C_{LNA}\to C_{DNA})=\Delta \Delta G_{solv}^{LNA,M\to H}+\Delta \Delta G_{solv}^{C,L\to D}=0.68\;kcal/mol$$

## Conclusions

We investigate the transformation between locked ribose and deoxyribose for pyrimidine nucleotides and the associated free energy change. To do that we developed two protocols for single and dual topology, using molecular dynamics simulation with the CHARMM36 force field and the BAR method.

The first approach for the transformation from LNA to DNA is divided in three‐steps: (1) the DNA angle and dihedral energies are turned on (*s*0), (2) the charges and the Lennard‐Jones parameters are mutated (*s*1), (3) the LNA angle and dihedral energies are turned off (*s*2). For the single topology, *s*0 can be omitted, and the main transformation is completed in *s*1. For the dual topology, *s*1 also involves the most significant energy change, but here *s*0 and *s*2 are nontrivial because the bonded terms change due to the conformational contribution in the bridged ring. The second approach is a one‐step approach, where bonded and non‐bonded terms are mutated in one step. It requires some careful adaptation in the treatment of angle terms to avoid deformation of the ring scaffold. This approach requires fewer windows than the three‐step approach, making it more efficient. Both approaches give similar results, *viz*. that the solvation free energy for an LNA nucleoside is 1–2 kcal/mol more favorable than for a DNA, because of the hydrophilicity of oxymethylene. We are not aware of any direct measurement of the solvation free energy of an LNA nucleoside, but $\Delta \Delta G_{solv}^{L\to D}$ is comparable with experimental data for cyclic and linear ether analogs.

Although most windows were run for 10 ns (20 ns for some in dual topology), 5 ns sampling time seems to be enough to get converged results for the three‐step and one‐step approaches in either topology, except for thymidine in the three‐step approach of dual topology, where longer sampling time is suggested for the windows near *λ_s_*_0_ = 1/*λ_s_*_1_ = 0. Both three‐step and one‐step protocols can be used, but the one‐step approach in dual topology is more complex to setup as it requires the user to be quite familiar with the force field parameters of the molecule.

## Supporting information

Supporting Information

Supporting Information

Supporting Information

Supporting Information

Supporting Information

Supporting Information
