# Supplementary material for: The free energy of locking a ring: Changing a deoxyribonucleoside to a locked nucleic acid
Source: J Comput Chem. 2017 Jan 19;38(15):1147–57. doi: 10.1002/jcc.24692 (PMC5434909; doi:10.1002/jcc.24692)
Supplement: Supplementary file 1 — Supporting Information [file JCC-38-1147-s001.pdf]

# The free energy of locking a ring: changing a deoxyribonucleoside to a locked nucleic acid

## Supporting information

You Xu, Alessandra Villa, Lennart Nilsson

Department of Biosciences and Nutrition, Karolinska Institutet, SE-141 83 Huddinge, Sweden

**Table S1**. Standard deviation ($\sigma$, kcal/mol) of ΔG, and minimal energy distribution overlap between adjacent all neighbor windows ($O_{min}$, %) for each transformation step related to the number of $\lambda$s in the calculations using BAR. Group #1 is the original window scheme, and in groups #2 - #5 the number of $\lambda$s was reduced, and $\Delta G$ was recalculated. Step *s0* in single topology was omitted. The smallest number of $\lambda$s required to achieve $\sigma$<0.2 kcal/mol and $O_{min}$ > 1% are shown in bold. The total minimial number of $\lambda$s in the multi-step protocol (in bold) is the sum of the required minimal number of $\lambda$s of each step.

| | | #1 | | | #2 | | | #3 | | | #4 | | | #5 | | |
|---|---|---|---|---|---|---|---|---|---|---|---|---|---|---|---|---|
| | | $\lambda$ | $O_{min}$ | $\sigma$ | $\lambda$ | $O_{min}$ | $\sigma$ | $\lambda$ | $O_{min}$ | $\sigma$ | $\lambda$ | $O_{min}$ | $\sigma$ | $\lambda$ | $O_{min}$ | $\sigma$ |
| Single topology | s*1* | 11 | 35 | 0.05 | 9 | 27 | 0.05 | 7 | 22 | 0.05 | 4 | 6 | 0.05 | **3** | **1** | **0.07** |
| | *s2* | 11 | 35 | 0.15 | 9 | 16 | 0.18 | 7 | 4 | 0.18 | **5** | **4** | **0.15** | 4 | 0.3 | 0.37 |
| | total | 22 | | 0.16 | 18 | | 0.19 | 14 | | 0.19 | 9 | | 0.16 | **8** | | **0.17** |
| Dual topology | *s0* | 11 | 37 | 0.09 | 9 | 27 | 0.10 | 8 | 18 | 0.12 | **6** | **2** | **0.08** | 5 | 0.2 | 0.33 |
| | *s1* | 11 | 54 | 0.06 | 9 | 42 | 0.07 | 7 | 25 | 0.07 | 5 | 6 | 0.06 | **3** | **2** | **0.11** |
| | *s2* | 15 | 28 | 0.06 | 13 | 15 | 0.05 | 10 | 10 | 0.05 | **7** | **3** | **0.09** | 5 | 0.5 | 0.12 |
| | total | 37 | | 0.12 | 31 | | 0.13 | 25 | | 0.15 | 18 | | 0.14 | **16** | | **0.16** |
| | One-step | 17 | 22 | 0.05 | 14 | 15 | 0.08 | 12 | 3 | 0.14 | 10 | 1 | 0.16 | **9** | **1** | **0.15** |

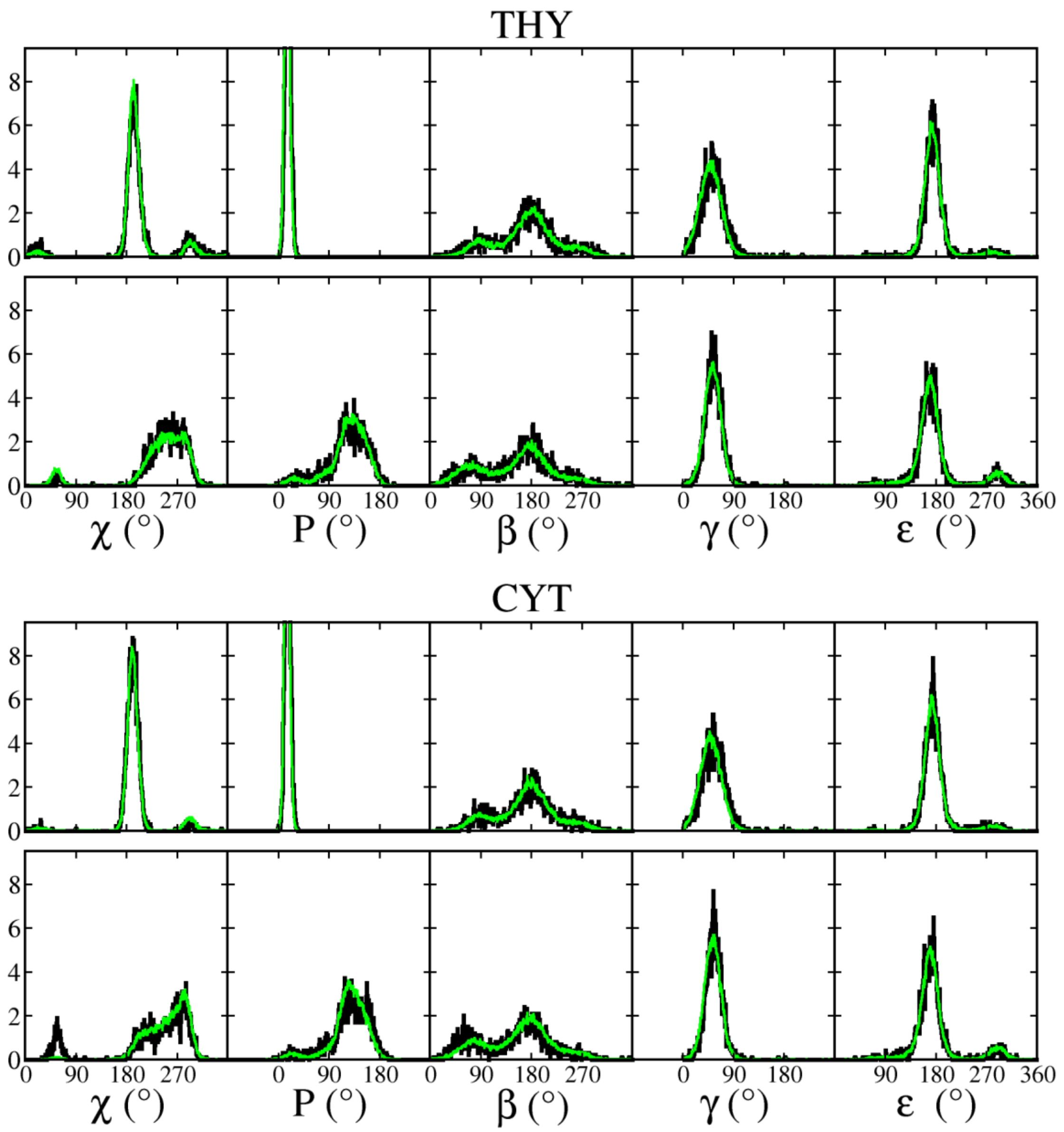


**Figure S1**. Conformational distributions of nucleosides T and C in the one-step protocol for the transformation LNA→DNA. The graph shows the glycosidic torsion (*χ*), sugar pucker (*P*) and backbone torsions *β*, *γ* and *ε*, in the initial (*λ*=0, LNA) and final (*λ*=1, DNA) states. The distributions are summed from five independent replicate simulations. For comparison the distributions sampled from 200 ns standard MD simulations of regular LNA and DNA nucleosides are shown in black.
